# YOLO12-SDS: an enhanced lightweight model for accurate and real-time fig maturity detection

**DOI:** 10.3389/fpls.2026.1957188

**Published:** 2026-09-01

**Authors:** Xiao Cui, Jiayi Li, Jisheng Liu, Yujin Guo, Miaomiao Zhang, Fuzhong Li, Xiaoying Zhang

**Affiliations:** Faculty of Software Technologies, Shanxi Agricultural University, Jinzhong, China

**Keywords:** computer vision, embedded deployment, fig, precision agriculture, ripeness detection

## Abstract

The contents of active ingredients such as isoprenyl phenols and the quality of figs are significantly regulated by ripeness. Accurate ripeness grading is critical for quality improvement and efficiency enhancement in the fig industry. However, existing detection methods suffer from poor environmental adaptability and excessive model redundancy, failing to simultaneously satisfy the requirements of high-precision, lightweight and real-time detection. To address the above limitations, this study takes YOLO12n as the baseline model and proposes a lightweight detection model named YOLO12-SDS for fig ripeness grading. Structurally, three customized modules are integrated into the proposed model. The SPDSPPF module adopts spatial rearrangement and multi-scale pooling fusion to strengthen the retention of small-object details and high-level feature extraction capability. The DySample module employs the PL-based learnable upsampling strategy to restore fruit contour, color and texture information with superior performance. The SCSA module leverages the collaborative spatial-channel attention mechanism to highlight critical ripeness-related features and suppress interference from complex backgrounds. Experimental results on the fig dataset demonstrate that compared with the original YOLO12n model, YOLO12-SDS achieves performance improvements of 2.1, 6.6, 4.8 and 1.0 percentage points in Precision, Recall, mAP@50 and mAP@50:95, respectively. The proposed model has a parameter count of 2.74 M, a computational cost of 6.0 GFLOPs and an inference speed of 115.6 FPS. Embedded deployment and testing are completed on the CUBE NANO platform. This study provides a technical solution with high precision, strong real-time performance and favorable deployment adaptability for fig ripeness grading in greenhouse facilities, which possesses great practical application value and broad promotion prospects.

## 1 Introduction

Figs are rich in bioactive secondary metabolites including flavonoids, coumarins and terpenoids, exhibiting strong antioxidant capacity and promising pharmaceutical exploitation value. Their unique isoprenyl phenolic constituents endow figs with distinctive functional properties (Wang et al., 2024c; Teruel-Andreu et al., 2021). Existing studies have clarified the physicochemical properties, accumulation patterns and synthetic pathways of active ingredients in figs, verifying their nutritional and medicinal values. By-products such as fruit flesh, leaves and seeds also display remarkable application potential in food processing and chronic disease intervention (Yang et al., 2023; Byeon and Lee, 2021). Lipidomic profiling has further revealed phenotypic diversity and differences in the nutritional characteristics of fig seed cultivars (Benyahia Irchad et al., 2023). Nevertheless, the synthesis, composition and content of isoprenyl phenols, as well as fruit antioxidant activity, physicochemical indicators and flavor quality, are all significantly modulated by ripeness (Lin et al., 2023). Accordingly, accurate determination of optimal harvest time and construction of an efficient ripeness discrimination system constitute the core approach to cutting postharvest losses and elevating the commercial value of figs.

Current research on figs has mainly focused on two major areas: biological mechanisms and postharvest management. Regarding biological mechanisms, Garza-Alonso et al. (2019) employed a hydroponic deficiency culture method to systematically investigate the effects of macronutrient deficiencies (nitrogen, phosphorus, potassium, calcium, and magnesium) on visual deficiency symptoms, vegetative growth, and mineral element contents in various organs of fig plants. They clarified the growth-inhibiting effects induced by individual element deficiencies, as well as the synergistic and antagonistic relationships among these elements. Jafari et al. (2018) grafted ‘Sabz’ fig scions onto five different rootstocks and, by measuring plant water-related parameters and leaf mineral element contents, revealed the regulatory roles of fig rootstocks on scion water status and nutrient accumulation under drought stress. In the field of postharvest research, Baigvand et al. (2015) utilized a machine vision system to automatically classify dried figs based on three key quality indicators (color, size, and cracking degree) combined with image processing algorithms. A dedicated classification program was developed using LabVIEW, enabling objective grading of dried fig quality. For fresh fig postharvest storage and logistics, Ertan et al. (2019) conducted low-temperature storage and shelf-life simulation experiments on multiple fresh fig cultivars. By measuring a series of physicochemical quality indices, they systematically analyzed the postharvest storage and transportation performance of fresh figs, as well as the storability and transportability of fruits at different maturity stages. Existing research on figs has primarily focused on plant physiological responses and postharvest quality regulation, while studies on intelligent identification of fruit maturity remain relatively weak, with only a few scholars having conducted preliminary explorations based on deep learning. Sheidaee and Bazyar (2025) constructed a white fig maturity classification model based on ResNet-50, achieving a classification accuracy of 94.07% on an augmented dataset. However, this method is only applicable to single-fruit detection and cannot adapt to the dense distribution and mutual occlusion of fruits in greenhouse conditions.

Furthermore, in the field of fruit and vegetable ripeness detection, deep learning models have achieved remarkable progress on crops including camellia oleifera fruits, tomatoes, peaches and blueberries, offering valuable references for intelligent fig ripeness detection. To solve the problem of camellia oleifera fruit identification in open orchards, Zhu et al. (2024) constructed YO-LM based on YOLOv7-tiny, which substantially improved the accuracy of ripeness recognition. Aiming at leaf and branch occlusion and variable illumination of greenhouse tomatoes, Chen et al. (2024b) and Hao et al. (2025) proposed MTD-YOLO and GSBF-YOLO respectively, boosting the detection accuracy and stability of models under complex environments. Moreover, the Peach-YOLO model proposed by You et al. (2026) occupies merely 5.0 MB storage space with an inference speed of 115.7 FPS, striking a balance between detection accuracy and deployment efficiency for peaches. The SDGD-YOLO designed by Zhao et al. (2026b) only contains 1.988 M parameters and can accurately identify densely clustered black raspberry fruits. Wu et al. (2025b) integrated an improved YOLOv8 with the ByteTrack multi-object tracking algorithm to develop STF-YOLO, which realized ripeness discrimination and quantity counting of blueberries in open orchards. The aforementioned studies provide references for fig ripeness detection in terms of lightweight feature extraction, multi-scale information fusion and adaptability to complex environments. Efficient feature extraction for low-resolution images and small objects has also been investigated to improve the preservation of fine-grained visual information (Sunkara and Luo, 2023). Nevertheless, large variations in fruit size, leaf-branch occlusion and dense overlapping fruits remain prominent challenges, leaving considerable room for improvement in the environmental adaptability and lightweight deployment of existing algorithms.

In addition to lightweight CNN based detection networks, Transformer detection frameworks and vision foundation models have also advanced the development of agricultural intelligent perception. Benefiting from global attention mechanisms, Transformer architectures can model long range contextual information in complex orchard environments and achieve competitive performance under dense fruit and occlusion scenarios (Wu et al., 2025a; Zhou et al., 2025). Vision foundation models such as SAM and Grounding-DINO leverage general prior knowledge to alleviate the problem of insufficient field annotated data, making them suitable for crop segmentation and fruit recognition tasks (Liu et al., 2026a; Lundqvist et al., 2026). Nevertheless, such models generally suffer from a large number of parameters and high inference overhead, which creates practical obstacles for direct deployment on embedded edge devices. Therefore, balancing detection accuracy, environmental robustness and edge deployment costs remains a critical challenge to be addressed urgently for intelligent fruit detection in real world orchard applications.

In the field of image enhancement and feature optimization, numerous deep learning based studies provide support for image quality improvement and feature extraction in complex scenarios. Zhang et al. (2026a) optimized image quality using multi-layer information fusion and self-organized stitching. Zhang et al. (2026b) enhanced local and global feature extraction capabilities through a multidimensional feature collaboration network. Wang et al. (2026) proposed a text image fusion diffusion model that effectively addresses image enhancement under data limited conditions, which can provide references for the preprocessing and feature optimization of complex field images of figs.

To tackle the dual bottlenecks of complex environmental interferences and limited computing power for embedded deployment in fig ripeness detection, this paper takes YOLO12n as the baseline model and proposes an improved model termed YOLO12-SDS. The SPDSPPF module is constructed by reconstructing the SPPF structure with the introduction of the SPD-Conv module (Liu et al., 2023), which boosts the extraction capability of high-level multi-scale features. The PL-mode DySample upsampling module (Liu et al., 2023) replaces the original upsampling layer to improve the restoration of detailed information during feature fusion. The SCSA attention mechanism (Si et al., 2025) is embedded prior to the small-object detection branch to strengthen target features and suppress background interferences. Taking Bojihong figs (Liang et al., 2025) cultivated in greenhouses as the research object, a field real-scene dataset is established. Ablation experiments and comparative analyses with mainstream lightweight models verify the effectiveness and superiority of YOLO12-SDS. Moreover, embedded deployment and practical measurement verification of the proposed model are completed on the Jetson Orin Nano platform.

## 2 Materials and methods

### 2.1 Data acquisition

**Figure 1:**
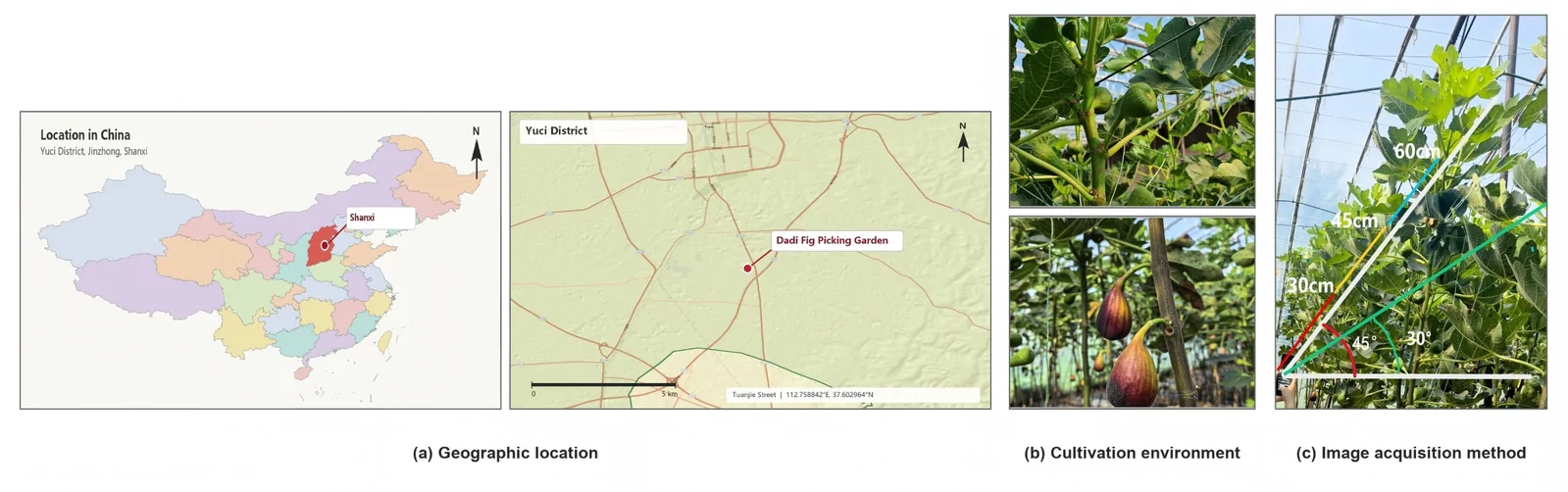
Dadi Fig Picking Garden, Yuci District, Jinzhong City, Shanxi Province.

Experimental data were collected from Dadi Fig Picking Garden in Yuci District, Jinzhong City, Shanxi Province, as shown in **Figure 1**, with Bojihong figs as the research material. The fig plants were cultivated in plastic arch greenhouses under protected cultivation and artificial pruning management. Three greenhouses were arranged in sequence. The middle greenhouse possessed superior heat-retention capacity with an overall higher indoor temperature and a smaller diurnal temperature difference, while the two side greenhouses had better ventilation conditions. The row spacing was 2.5–3.0 m, and the plant spacing was 1.2–1.5 m. Healthy fig plants with uniform growth vigor were randomly selected for image acquisition. Starting at approximately 22 days after fruit setting, images were captured every 3 days, covering the fruit expansion stage and ripening color-transition stage. An iPhone 16 Pro smartphone was used for shooting between 9:00 and 10:30 every day. Three shooting distances of 30 cm, 45 cm and 60 cm were set, and multi-angle oblique shooting at 30°–45° was adopted. Images under front lighting and backlighting conditions were collected simultaneously. The shooting scenarios covered complex greenhouse environments including sunny days, cloudy days, partial shading, branch and leaf occlusion, and overlapping fruits. A total of 3402 original images were finally obtained.

### 2.2 Dataset preprocessing

**Figure 2:**
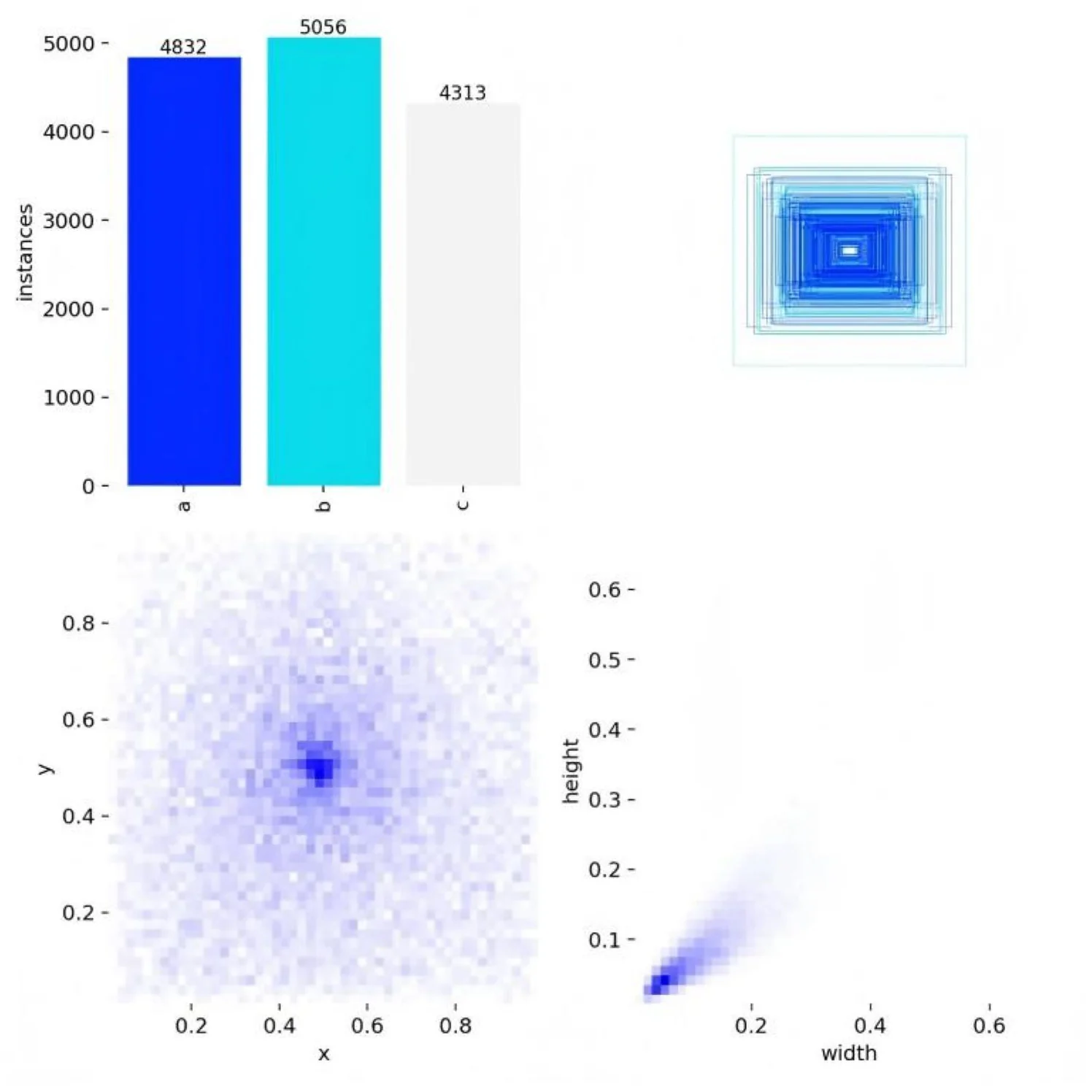
Analysis diagram of dataset label distribution.

Referring to the research on fruit ripening stage classification conducted by Pereira et al. (2020), combined with visual characteristics including fruit swelling degree and peel pigmentation of Bojihong figs, the ripeness levels were divided into three categories: immature, color-transition, and mature, which were labeled as a, b and c, respectively. Following the dataset labeling strategy for greenhouse detection proposed by Zhao et al. (2026b), LabelImg software was adopted to draw bounding boxes and export annotations in YOLO format. The label distribution of the dataset is shown in **Figure 2**. A total of 14,201 targets were annotated, consisting of 4,832 category a samples, 5,056 category b samples and 4,313 category c samples, indicating a balanced sample distribution across all categories. Small targets located at the central region of images dominated the dataset, which conformed to the small-object distribution rule. This dataset could reflect the field characteristics of complex target scales for figs grown in protected greenhouses. The whole dataset was randomly split at an 8:1:1 ratio to construct the training set, validation set and test set separately, ensuring the independence and scientific rationality of model training, parameter tuning and performance evaluation.

### 2.3 Methodology

**Figure 3:**
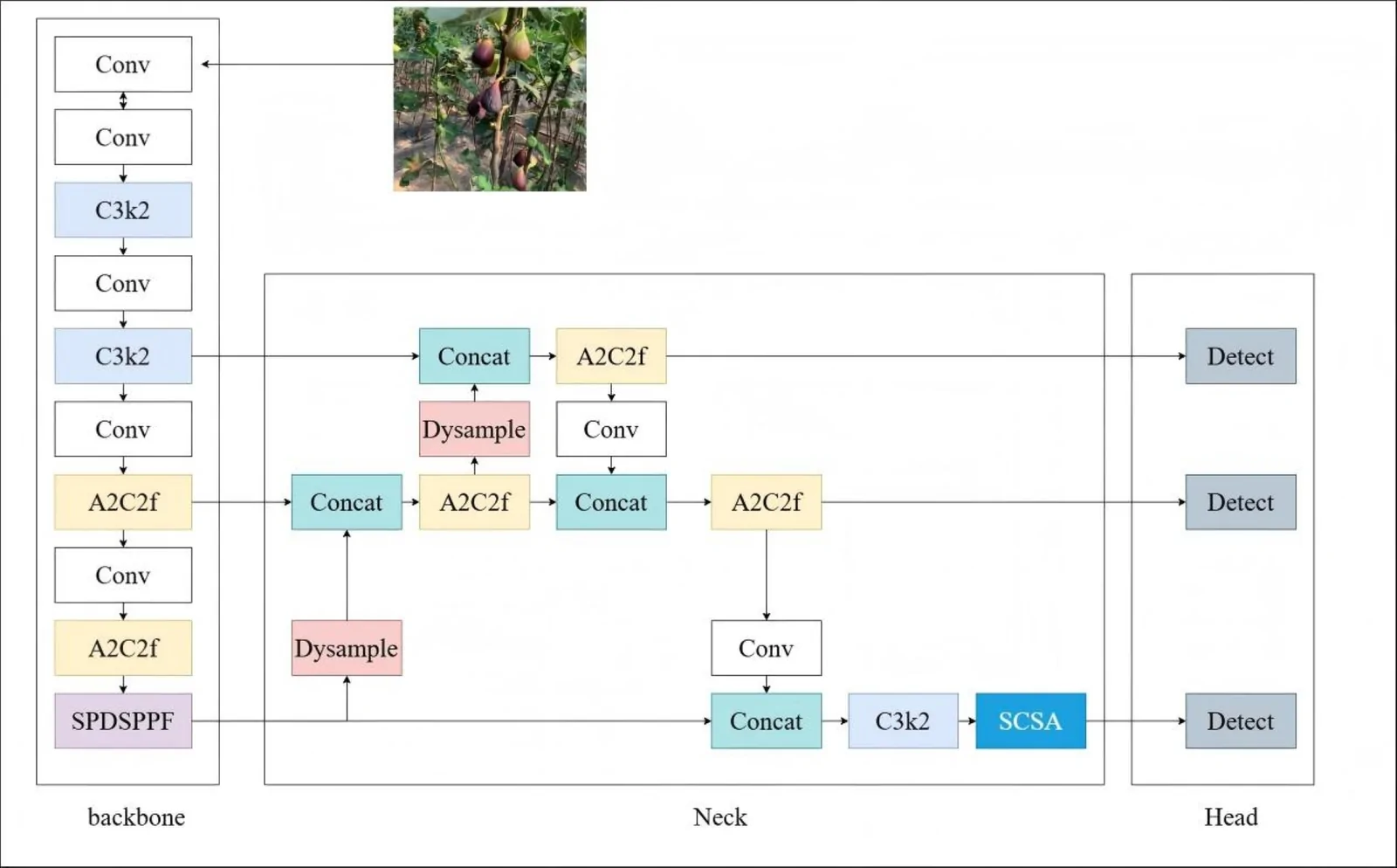
Architecture diagram of YOLO12-SDS.

As a lightweight one-stage detection network, YOLO12 adopts the A2C2f architecture and an efficient multi-scale feature fusion strategy, which reduces computational overhead while balancing feature transmission efficiency and real-time inference performance. Aiming at the challenges in greenhouse fig detection, including dramatic fruit scale differences, front-back inter-fruit occlusion, complex background noise, and low discriminability of ripeness features caused by uneven illumination inside greenhouses, this study takes YOLO12n as the baseline and proposes an improved detection model YOLO12-SDS, whose architecture is shown in **Figure 3**. On the basis of SPD-Conv, the SPDSPPF module is designed to enhance the retention of small-object features via spatial rearrangement and multi-scale pooling fusion. The PL-mode DySample learnable upsampling is applied to achieve fine restoration of fruit texture and ripeness-related features. Furthermore, the SCSA spatial-channel collaborative attention mechanism is embedded to suppress background interferences and focus on critical discriminative features of fruits.

#### 2.3.1 SPDSPPF

**Figure 4:**
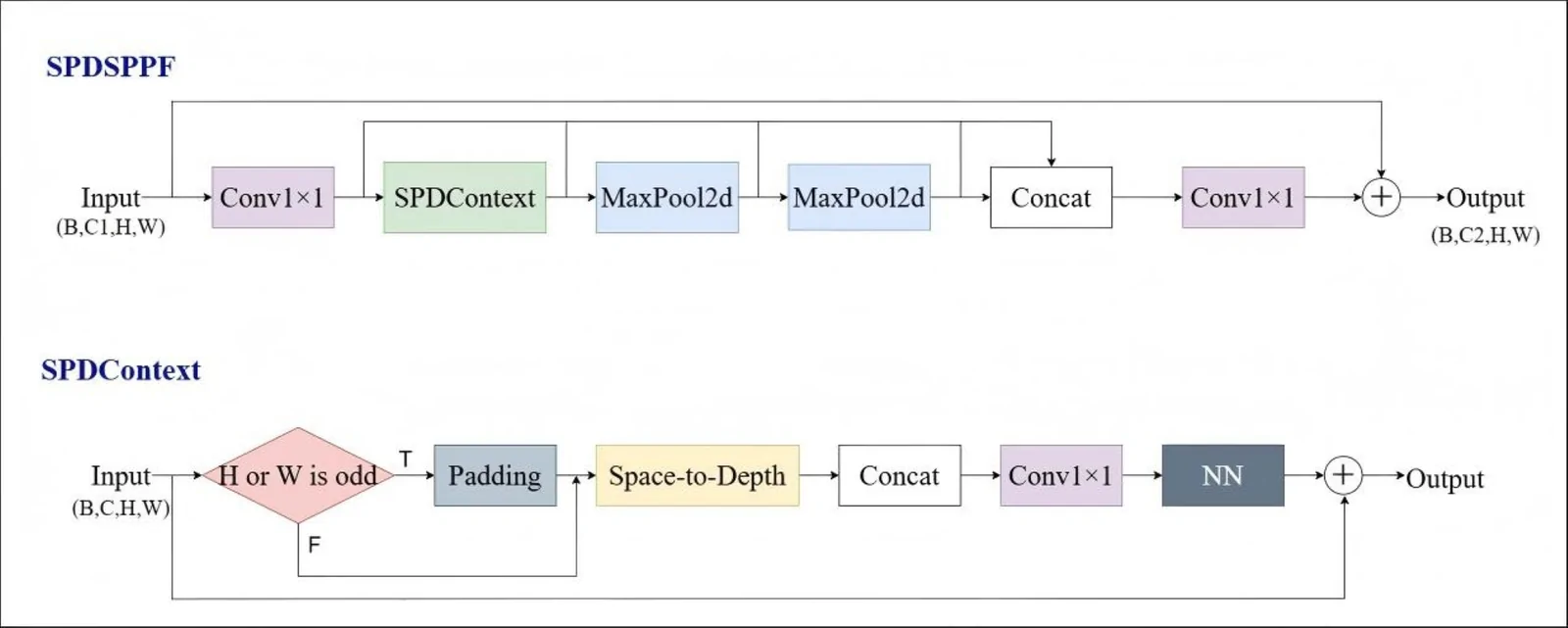
Structure diagram of SPDSPPF module.

The backbone network of YOLO12 adopts the A2C2f architecture to achieve efficient high-level feature extraction. Nevertheless, when applied to greenhouse fig detection scenarios, it suffers from insufficient multi-receptive-field feature fusion and severe attenuation of detailed features for tiny fruits and mutually occluded targets. To tackle this limitation, this study introduces the SPD spatial rearrangement mechanism and constructs the SPDSPPF enhancement module. Through space-to-channel feature rearrangement and multi-scale pooling fusion, the module enriches receptive field diversity and strengthens the preservation of fine-grained information in high-level features (Chen et al., 2024a; Li et al., 2026), thus improving the robustness of critical fig features against complex backgrounds. The detailed structure is shown in **Figure 4**.

Taking input channels as the baseline, this module first compresses the number of channels to half of the original via 1×1 convolution. Afterwards, a space-to-channel feature rearrangement structure reorganizes spatial information within the 2×2 neighborhoods of feature maps into the channel dimension for fusion, thereby preventing detail loss caused by conventional pooling operations. Two successive max-pooling layers with a kernel size of 5, stride of 1 and padding of 2 are subsequently applied to construct multi-branch receptive field features. Finally, multi-path features are concatenated, followed by a 1×1 convolution to restore the feature channels to the target output dimension. The module adopts a kernel size k=5 with a total of three feature branches, and residual skip connections are introduced to fuse original information and enhanced features, achieving more robust multi-scale feature representation with low computational overhead. In the YOLO12-SDS model, the SPDSPPF module is embedded after the output of the last A2C2f layer at the end of the backbone network. It takes 1024-channel high-level semantic features as input. After internal channel compression, spatial rearrangement and multi-branch pooling fusion, it still outputs 1024-channel features, with residual connections enabled to realize identity mapping. This embedding strategy does not alter the original feature transmission path or scale structure of the network, while further strengthening the model’s capacity to retain detailed features of tiny fig fruits and occluded fruits. It provides richer, more discriminative high-level semantic information for subsequent neck multi-scale feature fusion and detection head classification.

#### 2.3.2 DySample

**Figure 5:**
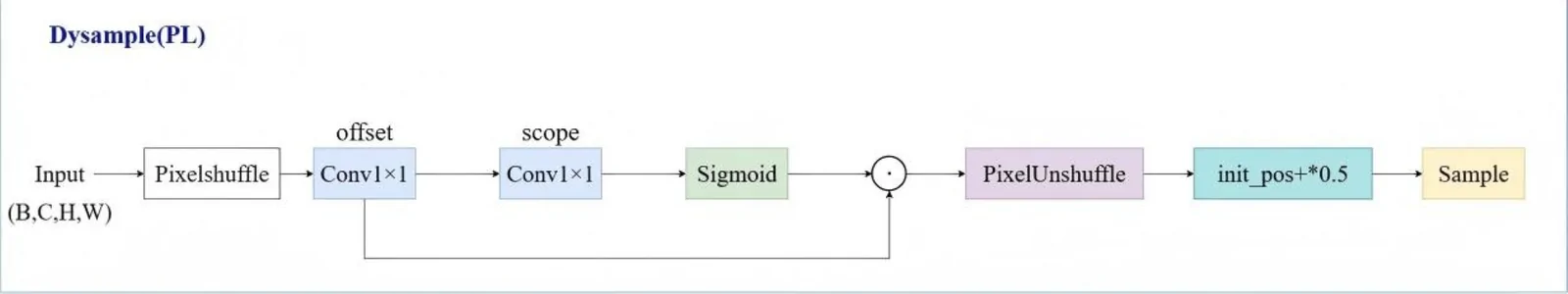
Structure of DySample module.

Traditional fixed-interpolation upsampling delivers high efficiency and conciseness during the feature fusion phase of YOLO12. However, its non-adaptive sampling strategy leads to insufficient fine-grained reconstruction of fig edge contours and ripeness-related texture features (Liu et al., 2026b). To address this defect, this study introduces the learnable upsampling unit DySample and constructs an adaptive upsampling module under the PL mode. Relying on a mechanism that reorganizes channels before offset sampling, the module achieves high-precision upsampling of low-resolution features (Zhang et al., 2025) and significantly improves the reconstruction accuracy of fruit edges and peel color features. The structure of this module is shown in **Figure 5**.

Based on an upsampling factor of 2, under PL mode this module first transforms the channel and spatial dimensions of input features via pixel rearrangement operations. Then 1×1 convolutions are applied to learn multi-dimensional offset weights, and a grouped sampling strategy is adopted to realize adaptive positioning of feature grids. The module is configured with four groups, where each offset generation branch centers on 1×1 convolution. Fixed initialization is utilized to guarantee sampling stability, enabling refined feature upsampling without introducing excessive parameters.

In the YOLO12-SDS model, the DySample module replaces the two original fixed upsampling layers in the network neck. It performs 2× upsampling on middle-level features with 512 channels and 256 channels separately, and both the channel number and feature scale are strictly matched with the subsequent concatenation structures. This design retains more detailed information of figs during multi-scale feature fusion, and further improves the model’s feature utilization efficiency for tiny targets and occluded fruits.

#### 2.3.3 SCSA

**Figure 6:**
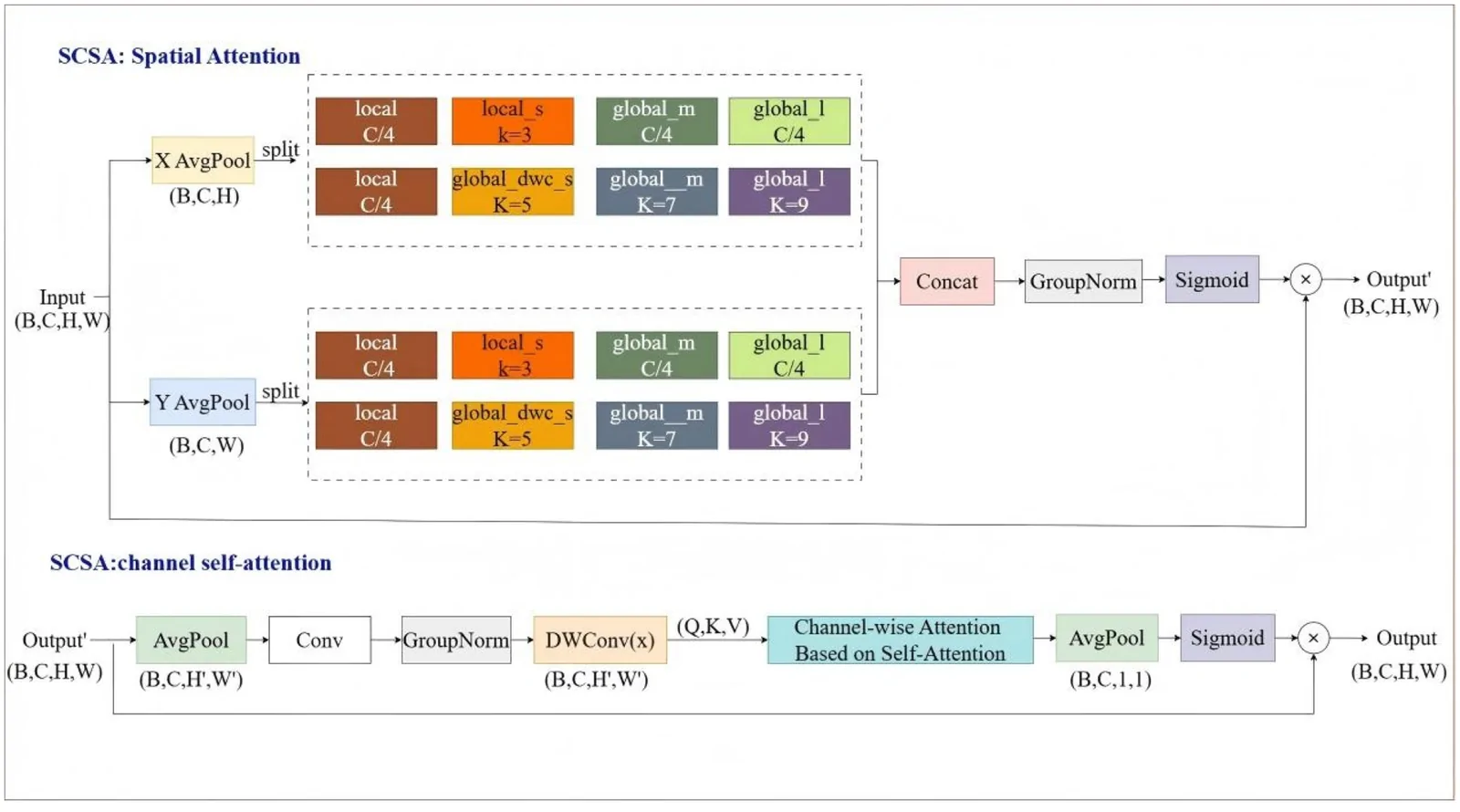
Structure of the SCSA joint attention mechanism.

Under complex greenhouse environments, background information such as leaves and branches is mixed with fig features. Relying solely on the original feature representation of YOLO12 fails to achieve precise focusing and enhancement of critical ripeness-related target features. To solve this problem, this study introduces the collaborative SCSA attention mechanism. With dual enhancement strategies including multi-scale spatial perception and self-attentive channel modeling (Wang et al., 2024b, 2023), the mechanism concentrates on valid fruit features while suppressing interfering background information, thereby boosting the model’s feature discrimination capability under complicated scenarios. Its architecture is shown in **Figure 6**.

This module splits input features into multiple branches, where one-dimensional depthwise convolutions with different receptive fields are applied to capture local and global spatial dependencies respectively. Spatial feature screening is realized through channel-wise weighting. On the channel branch, based on grouped self-attention mechanisms, query, key and value features are generated after dimension reduction via downsampling, followed by the calculation of attention weights. Feature enhancement is finally completed through global weighting along the channel dimension. The module automatically assigns the number of attention heads according to input channels by default, and four sets of convolution kernels with distinct sizes are utilized to implement multi-scale spatial perception. It elaborately models feature correlations while maintaining computational efficiency.

In the YOLO12-SDS model, the SCSA module is embedded at the front end of the P3 layer within the small-object detection branch. It performs attention enhancement on shallow high-resolution features with 256 channels while keeping the number of output channels and the size of feature maps unchanged. This location design directly strengthens the detailed and ripeness-related features of tiny fig fruits and effectively suppresses complex background interferences, supplying purer and more discriminative feature inputs for the small-object detection head.

### 2.4 Experimental environment and evaluation metrics

All experiments were conducted on the Windows 10 operating system, with hardware equipped with an NVIDIA GeForce RTX 4080 GPU and 32 GB RAM. The software environment was built based on the PyTorch 2.2.0 framework, CUDA 11.8, MMCV 2.2.0 and NumPy 1.26.4. During model training, the batch size was set to 12, the number of data loading workers to 6, and the maximum training epochs to 250. Stochastic Gradient Descent (SGD) was adopted as the optimizer. The initial learning rate lr0 was set to 0.001, the final learning-rate scaling factor lrf was set to 0.01, the momentum of the SGD optimizer was set to 0.937, and the weight decay coefficient was set to 0.0005. Data augmentation strategies including Mosaic augmentation, random horizontal flipping, and HSV color jitter were adopted during the training process.

To comprehensively evaluate the detection performance and computational efficiency of all models, analyses were carried out from two dimensions: detection accuracy and real-time performance. Precision (P), Recall (R), mAP@50 and mAP@50:95 were selected as the evaluation metrics for detection accuracy. Meanwhile, Parameters, GFLOPs and FPS (Frames Per Second) were used to quantify model complexity and inference speed, so as to comprehensively assess the deployment feasibility of models on edge devices (Wang et al., 2024a).

## 3 Experimental results and analysis

### 3.1 Overall experimental results

To guarantee the objectivity and generalization ability of experimental results and reduce experimental errors caused by random dataset splitting, five-fold cross-validation was adopted for the training and testing processes of all models in this study. The overall detection performance of the models and category-specific metrics are presented in **Table 1**, where category a refers to immature figs, category b to partially ripe figs, and category c to fully mature figs.

**Table 1:** Summary of experimental results.

Classes	P	R	mAP@50	mAP@50:95	Parameters	GFLOPs	FPS
All	0.817	0.807	0.879	0.715	2,741,137	6.0	115.6
a	0.781	**0.826**	0.882	0.726			
b	0.821	0.773	0.871	0.684			
c	**0.849**	0.822	**0.884**	**0.735**			

Bold values represent the optimal results in each group.

It can be concluded from the overall metrics that the proposed YOLO12-SDS model achieves a favorable trade-off between detection accuracy and lightweight characteristics, and exhibits stable grading and recognition performance under complex background interferences in greenhouses. Analysis of category-wise performance differences reveals that mature fruits possess high feature discriminability and intact morphology with low susceptibility to occlusion and illumination disturbances, thereby delivering the best detection performance. Partially ripe figs feature gradual color transition and blurry feature boundaries, leading to weak distinguishability and relatively lower overall detection accuracy. Immature fruits are mostly small-scale targets, yet they achieve outstanding Recall values, which demonstrates that the model has favorable perception capacity for weak-feature and tiny objects and can effectively mitigate the risk of field missed detection. Meanwhile, the model boasts low overall parameter count and computational overhead, with an inference speed of 115.6 FPS, which satisfies the application requirements of real-time detection and grading in protected agriculture.

### 3.2 Ablation experiments

To verify the effectiveness of the three improved modules (SPDSPPF, DySample and SCSA) one by one as well as their synergistic gain relationships, multiple groups of controlled ablation experiments were designed in this study, and the experimental results are presented in **Table 2**.

**Table 2:** Summary of ablation experimental results.

Model	Module			P	R	mAP@50	mAP@50:95	Parameters	GFLOPs	FPS
	A	B	C							
1				0.796	0.741	0.831	0.705	**2,508,929**	**5.8**	**142.4**
2	✓			0.773	0.76	0.817	0.663	2,739,329	6.0	138.6
3		✓		0.750	0.753	0.830	0.679	2,509,713	**5.8**	126.7
4			✓	0.749	0.798	0.859	0.694	2,509,953	**5.8**	134.9
5	✓	✓		0.726	**0.812**	0.823	0.662	2,751,681	6.0	129.8
6	✓		✓	**0.853**	0.758	0.832	0.673	2,740,353	6.0	128.4
7		✓	✓	0.809	0.775	0.849	0.710	2,510,737	**5.8**	121.3
8	✓	✓	✓	0.817	0.807	**0.879**	**0.715**	2,741,137	6.0	115.6

Bold values represent the optimal results in each group.

The ablation experimental results demonstrate that each individual improved module delivers performance improvements with distinct focuses on the baseline model, and each achieves targeted metric gains. When the SCSA module is introduced alone, the model obtains the most prominent gains in Recall and average precision, which effectively alleviates the missed detection of targets under complex field backgrounds. After embedding the DySample module independently, the multi-scale detection accuracy of the model is slightly optimized, and the model gains better adaptability to fine-grained features. When the SPDSPPF module acts alone, the overall model performance fluctuates slightly while its basic detection capacity remains stable. The two-module combination experiments reveal complementary performance effects among different improved modules. Compared with single-module optimization, module combinations integrate multi-dimensional optimization merits. Among all combinations, the pairing of DySample and SCSA yields the most remarkable enhancement to the comprehensive detection performance of the model.

After the collaborative embedding of all three modules, the model achieves optimal comprehensive detection performance. Compared with the baseline model, mAP@50 is increased by 4.8 percentage points, and mAP@50:95, which reflects the integrated accuracy for both large and small targets, is improved simultaneously. This verifies that the three improved structures deliver obvious synergistic gains. The model’s parameter quantity and computational complexity only experience slight growth, and the inference speed remains at the level for real-time detection. It demonstrates that the proposed improved strategy can effectively boost detection accuracy while restraining model complexity, and possesses excellent lightweight optimization characteristics.

### 3.3 Model robustness verification

**Figure 7:**
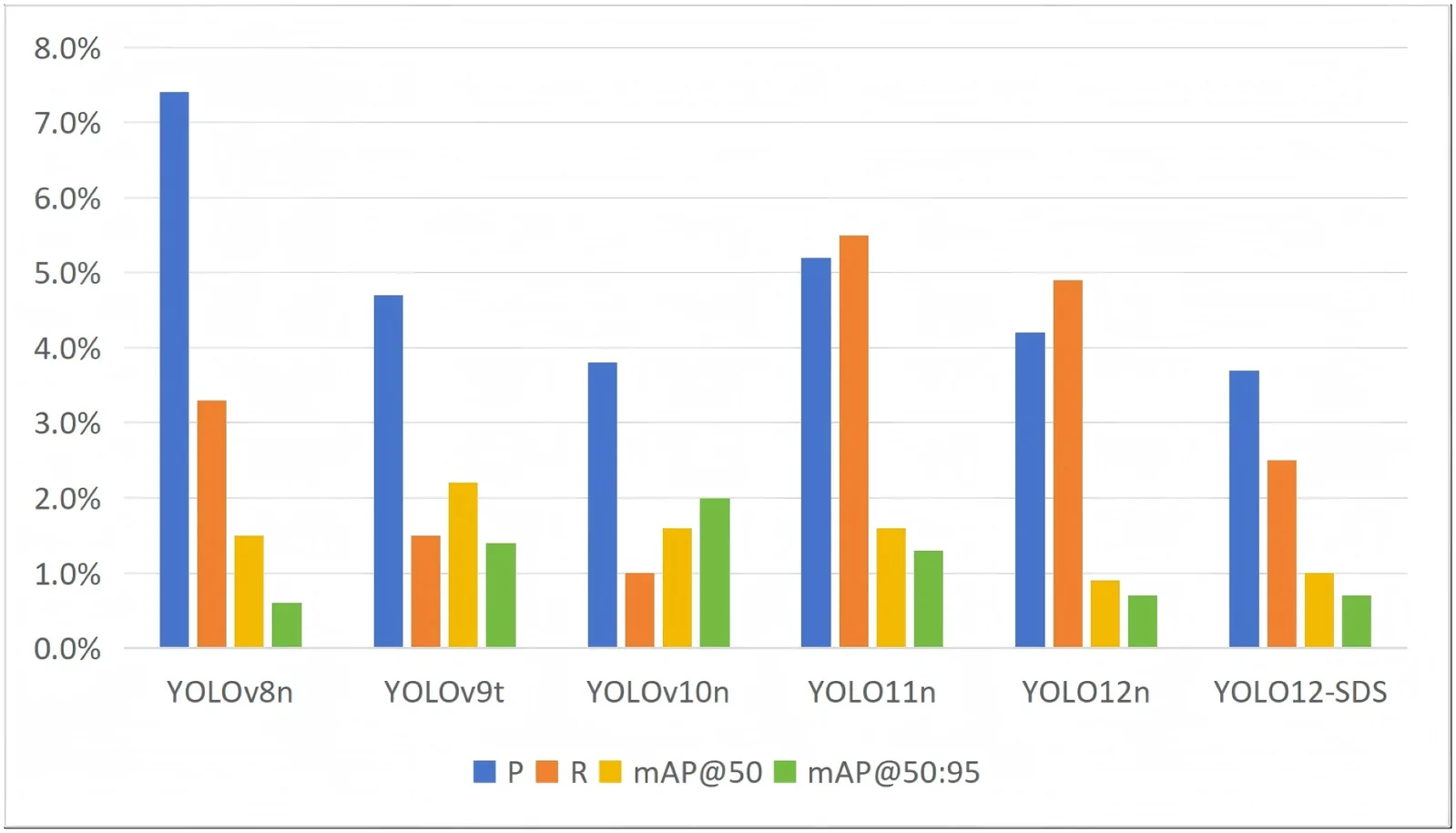
Comparison of indicator standard deviations among different lightweight detection models.

To verify the stability and experimental reproducibility of the model’s detection performance, a five-fold cross-validation strategy was adopted in this study. For each fold of data partitioning, three different random seeds were set for repeated training and testing. The sample standard deviation of key detection indicators was calculated for each model to quantify the performance fluctuation under the disturbances of data partitioning and training initialization differences. The indicator fluctuations of each model are shown in **Figure 7**.

It can be observed that the comparison models exhibit varying degrees of fluctuation in some indicators, and are particularly sensitive to data partitioning and training initialization in terms of precision. The proposed YOLO12-SDS model maintains relatively smaller fluctuations in this indicator, with better stability than the other comparison models. The overall fluctuation of the recall rate is moderate; although the improved model does not achieve the minimum fluctuation in this indicator, it still remains in a low fluctuation range with stable and reliable performance. The overall fluctuation levels of mAP@50 and mAP@50:95 are relatively low, among which YOLO12-SDS presents outstanding fluctuation control performance. The standard deviations of both indicators are controlled within 1%, ensuring highly consistent performance output across multiple repeated experiments. Overall, there are certain differences in the fluctuation ranges of indicators among various models. The proposed model exhibits smaller performance variations on key detection indicators and demonstrates more robust overall performance.

### 3.4 Comparative experiments

#### 3.4.1 Multi-model comparative experiments

To verify the overall competitiveness of the YOLO12-SDS model, comparative experiments were conducted between the proposed model and mainstream lightweight models including YOLOv8n, YOLOv9t, YOLOv10n, YOLO11n and the original YOLO12n under identical datasets and training hyperparameters. In addition, lightweight dedicated models YOLO11-SCC (Zhao et al., 2026a) and YOLO11-WBO (Zhang et al., 2026c) for greenhouse strawberry and blueberry detection were adopted. RT-DETR with Transformer architecture was also included as a representative non-YOLO model. The experimental results are presented in **Table 3**.

**Table 3:** Summary of multi-model comparative experimental results.

Model	P	R	mAP@50	mAP@50:95	Parameters	GFLOPs	FPS
YOLOv8n	0.754	0.763	0.804	0.651	3,006,233	8.1	**244.1**
YOLOv9t	0.776	0.796	0.817	0.693	**1,971,369**	7.6	92.7
YOLOv10n	0.740	0.729	0.758	0.647	2,695,586	8.2	166.0
YOLO11n	0.801	0.772	0.825	0.709	2,582,737	6.3	187.1
YOLO12n	0.796	0.741	0.831	0.705	2,508,929	**5.8**	142.4
RT-DETR	0.787	0.773	0.806	0.683	32,009,432	103.9	64.2
YOLO11-SCC	0.816	0.781	0.843	0.706	2,619,994	6.7	42.2
YOLO11-WBO	**0.823**	0.776	**0.852**	**0.717**	3,553,934	6.6	134.2
YOLO12-SDS	0.817	**0.807**	0.879	0.715	2,741,137	6.0	115.6

Bold values represent the optimal results in each group.

Experimental results reveal that YOLO12-SDS outperforms all comparison models in all core detection metrics including Precision, Recall, mAP@50 and mAP@50:95, and delivers superior feature discrimination capability for the fine-grained classification task of fig ripeness levels. As greenhouse fruit-vegetable detection models, YOLO11-SCC and YOLO11-WBO achieve favorable detection accuracy by virtue of causal attention and semi-supervised learning. Nevertheless, YOLO11-SCC only reaches an inference speed of 42.2 FPS, which makes it difficult to meet the requirements of mobile side deployment; YOLO11-WBO has a parameter count of 3.55 million with relatively high computational cost, and both models yield lower mAP@0.5 values than YOLO12-SDS. Transformer based RT-DETR achieves acceptable detection accuracy, yet it contains 32 million parameters with 103.9 GFLOPs and delivers an inference speed of merely 64.2 FPS. Its substantial computational overhead hinders deployment on low power orchard edge devices.

In terms of model complexity, the parameter count and computational overhead of the proposed model are slightly higher than ultra-lightweight counterparts such as YOLO12n and YOLO11n, yet markedly lower than YOLOv8n and YOLOv10n, maintaining an overall lightweight network architecture. Although the inference speed of the proposed model experiences a minor drop compared with some ultra-fast inference models, it still achieves real-time inference performance exceeding 115 FPS, which fully meets the deployment requirements of field equipment. The trade-off between comprehensive accuracy and model complexity demonstrates that YOLO12-SDS possesses stronger capacity to distinguish fine-grained features of figs at different ripeness stages. It can well adapt to grading and detection scenarios with drastic variations in fruit morphology and peel color, and thus exhibits better comprehensive practical performance than existing mainstream lightweight detection models.

#### 3.4.2 Comparative experiments of multi-level attention mechanisms

To explore the optimal embedding position of the SCSA attention module across multi-scale feature layers, comparative experiments of single-level and multi-level combined embedding were carried out on the shallow P3 layer, middle P4 layer and deep P5 feature layers based on the YOLO12-SDS model. The experiments analyzed the optimization effects of the attention mechanism on fig features of various scales, and the corresponding results are presented in **Table 4**.

**Table 4:** Summary of comparative experiments on multi-level attention mechanisms.

Model	Level			P	R	mAP@50	mAP@50:95
	P3	P4	P5				
1	✓			**0.817**	0.807	**0.879**	**0.715**
2		✓		0.773	**0.827**	0.854	0.693
3			✓	0.789	0.764	0.817	0.650
4	✓	✓		0.762	0.794	0.828	0.650
5	✓		✓	0.752	0.818	0.865	0.708
6		✓	✓	0.771	0.757	0.845	0.655
7	✓	✓	✓	0.794	0.778	0.877	0.706

Bold values represent the optimal results in each group.

The experimental results demonstrate that the embedding layer and combination form of the SCSA attention module exert a significant influence on the model’s detection performance. The model achieves the best comprehensive detection performance when the attention mechanism is only introduced into the shallow high-resolution P3 feature layer. Shallow features contain abundant detailed and tiny-object information, and attention enhancement can effectively strengthen the feature representation of occluded and small-scale fruits, thus improving fine-grained detection capability. Embedding the attention module solely in the middle P4 layer or deep P5 layer triggers performance degradation to varying degrees. Deep features carry highly abstract semantic information, and additional attention constraints tend to break the original feature mapping relationships, resulting in reduced detection accuracy.

The experimental results of multi-layer superimposed embedding reveal that dual-layer and triple-layer attention combinations fail to bring performance gains and generally lead to declined metrics. Superposition of multi-scale attention easily results in redundant feature constraints, which causes unnecessary feature suppression and information interference and reduces the network’s efficiency in focusing on valid target features. Accordingly, the optimal embedding strategy is to deploy the SCSA attention module solely on the shallow P3 feature layer. This scheme retains fruit detailed features while effectively suppressing background noise, making it suitable for the detection task of figs under complex greenhouse scenarios.

## 4 Discussion

### 4.1 Lightweight deployment

**Figure 8:**
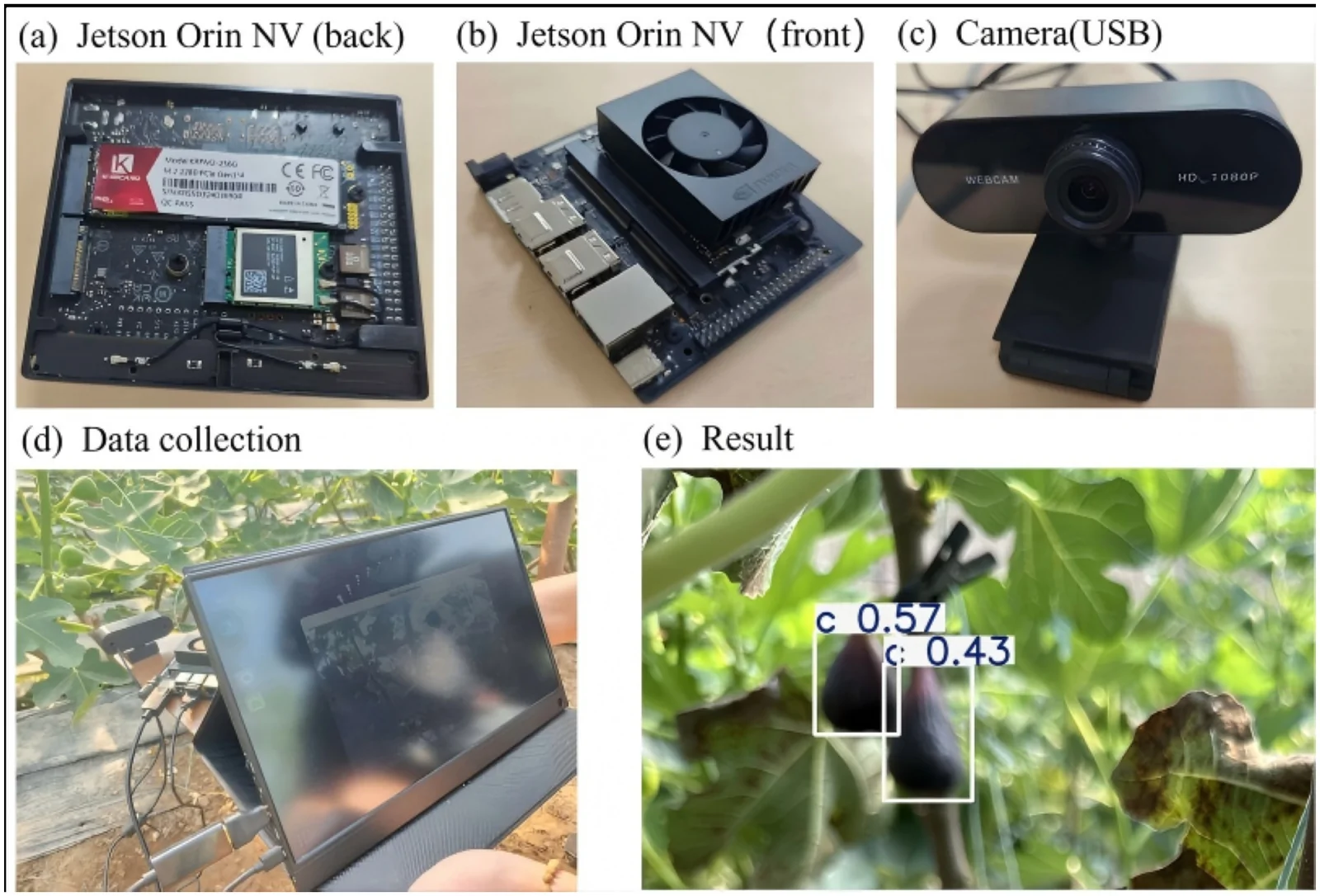
Field edge deployment system for fig maturity detection. **(a)** Jetson Orin NV (back); **(b)** Jetson Orin NV (front); **(c)** Camera (USB); **(d)** Data collection; **(e)** Result.

To verify the feasibility of field edge deployment of the YOLO12-SDS model, an NVIDIA Jetson Orin Nano SUPER 8 GB embedded development board was used to build a field test platform, as shown in **Figure 8**. This platform delivers an AI inference performance of 67 TOPS, a memory bandwidth of 102 GB/s and 8 GB RAM. It balances low power consumption and inference speed, making it suitable for field detection applications in protected agriculture. The YOLO12-SDS model has a parameter count of 2.74 M and computational cost of 6.0 GFLOPs. With outstanding lightweight characteristics and low hardware resource consumption, its computational requirements are fully compatible with the computing capacity of this development board.

The model achieves an inference speed of 115.6 FPS on desktop hardware. After being transplanted to the embedded device, its inference speed decreases due to limitations of hardware computing power and system scheduling. The measured average single-frame inference latency is 9.4 ms, which still meets the basic requirements for real-time detection and can be applied to tasks including real-time field video stream sampling and fig ripeness grading detection. In summary, YOLO12-SDS integrates the advantages of low computing overhead and high detection accuracy, and exhibits strong adaptability to the Jetson Orin Nano SUPER 8 GB edge platform. It possesses practical deployment value and promotion potential for intelligent detection in protected agriculture.

### 4.2 Visualization analysis

**Figure 9:**
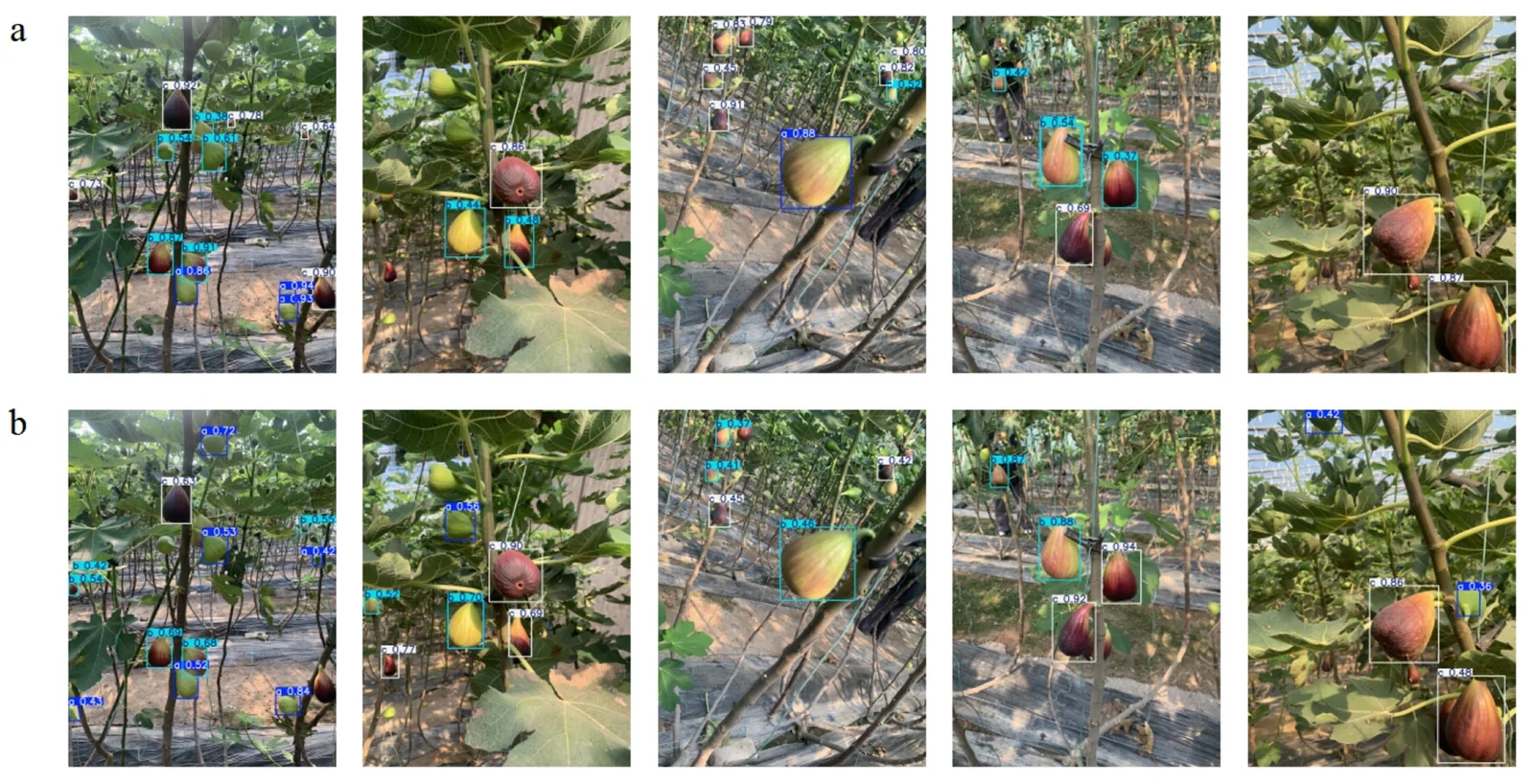
Comparison of fig detection results of different models under complex backgrounds. **(a)** YOLO12n, **(b)** YOLO12-SDS.

To intuitively compare the actual detection performance of the original YOLO12 and the proposed YOLO12-SDS models, five groups of challenging images with complex backgrounds were selected for visualized comparative analysis, and the results is shown in **Figure 9**. In the first group of images, the improved model missed a small number of figs in the central area, yet it achieved superior category recognition accuracy compared with the baseline model. For the second group, the improved model failed to detect the immature fruit at the top blended into the green background; this target bears high similarity to surrounding vegetation and is prone to missed detection. Nevertheless, the improved model successfully identifies more hard-to-detect targets with more precise category classification overall. In the third and fourth image groups, the improved model generated no false predictions for the largest central target in each frame. In the fifth group, the proposed model accurately recognized immature figs only partially exposed via their stalks, as well as the topmost immature fruit partially occluded by leaves. In conclusion, although minor missed detections still exist with the improved model, its overall missed detection rate and false detection rate are both lower than those of the original YOLO12. It delivers markedly stronger recognition performance for challenging fruits including occluded specimens and tiny-scale targets.

### 4.3 Quantitative analysis of detection errors and typical failure samples

**Figure 10:**
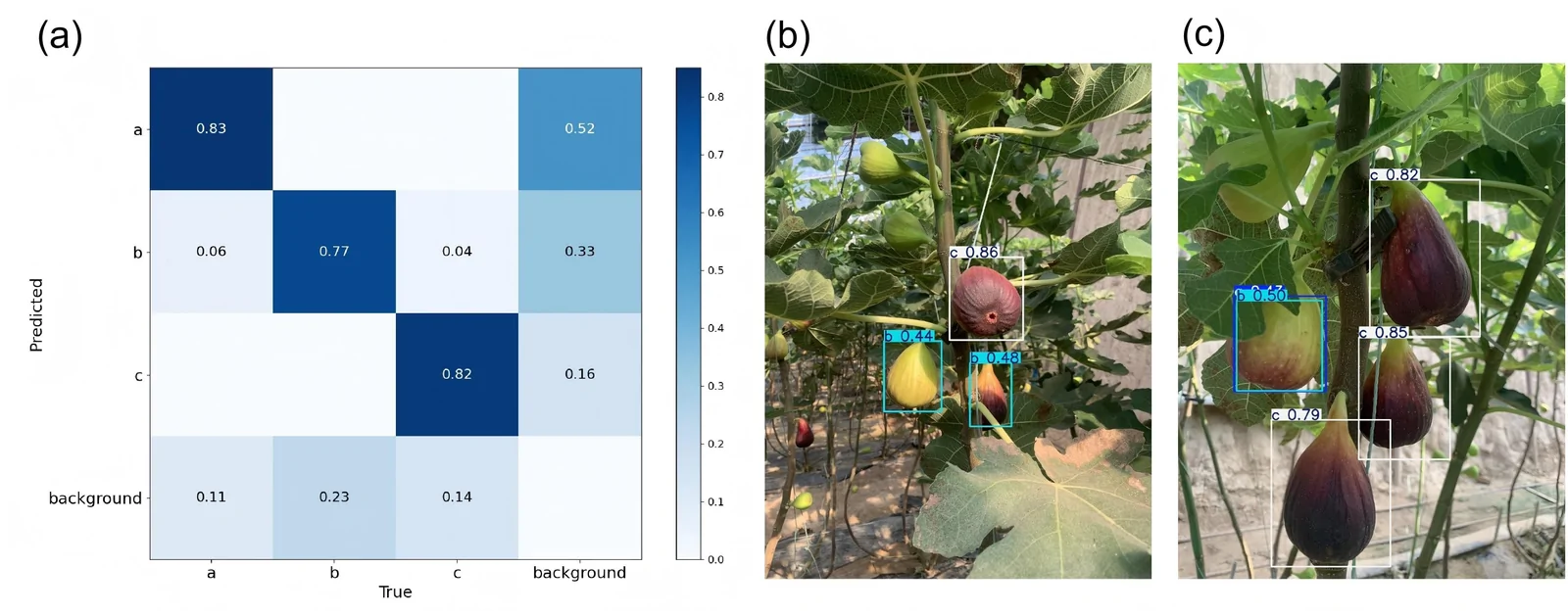
Quantitative analysis of model misclassification results and visualization of typical failure cases. **(a)** Normalized confusion matrix; **(b)** missed detection of immature fruits caused by branch and leaf background interference; **(c)** misclassification of fruits at the maturity transition stage.

To further analyze the recognition performance of the model for figs at different maturity stages and color transition samples, combined qualitative and quantitative analyses were carried out using the normalized confusion matrix and typical failure cases, as shown in **Figure 10**. **Figure 10a** presents the normalized confusion matrix, where the horizontal and vertical axes represent the predicted category and ground truth label, respectively. Labels a, b and c correspond to immature, color turning and mature fruits, and background denotes greenhouse interferences such as branches and leaves. The diagonal elements indicate that the immature class a achieves the highest recognition accuracy, followed by the mature class c, while the color turning class b obtains the lowest accuracy. Misclassifications mainly occur between transitional fruits and the background: 52 % of background regions are misclassified as immature fruits (a), and 33 % are misclassified as color turning fruits (b); meanwhile, 23 % of real color turning samples are missed and classified as background.

**Figures 10b, c** show two representative failed detection examples, which intuitively verify the error patterns revealed by the confusion matrix. In **Figure 10b**, the target fig is occluded by surrounding leaves. Affected by the complex branch and leaf background, the model fails to detect the immature fig in the image. The fruit in **Figure 10c** lies in the transition stage between immaturity and the color turning period, with gradually changing peel color and blurred boundaries of maturity features, which eventually leads to maturity misclassification. The above detection failures stem from the complex working conditions inside the greenhouse. The peel color of figs at the color turning stage presents a gradual change characteristic. Under uneven illumination and leaf occlusion, the visual features of fruits are highly similar to those of surrounding branches and leaves. Overall, YOLO12-SDS can effectively distinguish the three maturity classes with obvious feature differences, whereas its recognition capability for samples with severe background interference and gradual color transition still needs further optimization.

### 4.4 Cross scene generalization ability verification

Figs are shrubs that can be cultivated after dwarfing treatment, while blueberries are typical shrubs. The two crops exhibit significant differences in plant type, fruit morphology and canopy structure. It is reasonable to conduct cross scene verification using the blueberry dataset, which can effectively test the generalization performance of the model. To further evaluate the cross scene transfer ability of the model, this study adopts a dataset of greenhouse potted blueberries for external validation (Zhang et al., 2026c). The original dataset classifies blueberry samples from the late expansion stage to the color turning stage into five maturity grades. In this research, images of the color turning stage are extracted and relabeled into three categories, where the two intermediate maturity grades within the original color turning phase are merged. Experimental results show that the Precision, Recall, mAP@0.5 and mAP@0.5:0.95 of YOLO12n are 0.817, 0.849, 0.885, 0.767, while the corresponding metrics of YOLO12-SDS are 0.844, 0.865, 0.908, 0.784. Precision rises by 2.7%, Recall rises by 1.6%, mAP@0.5 rises by 2.3%, and mAP@0.5:0.95 rises by 1.7%. The improved model still retains competitive performance when both crop type and facility cultivation scenario are replaced. This result indicates that the proposed improvement strategy can effectively enhance the robustness of model feature extraction.

### 4.5 Limitations and future prospects

Combining the visualized detection results and edge deployment tests, the proposed YOLO12-SDS model still has certain application limitations. First, the model presents inconsistent detection performance across different fruit maturity stages. It is prone to misclassification and missed detection for color turning fruits with gradual peel color features under leaf occlusion and uneven illumination, as ambiguous maturity boundaries and complex backgrounds weaken the model’s fine grained feature extraction capability for challenging samples. Second, the dataset used in this study only covers conventional greenhouse illumination and weather conditions, without samples of extreme weather such as heavy rain, intense backlight and haze. Accordingly, the model has weak generalization capability under complicated environmental conditions. Third, after being transplanted to embedded devices, the inference frame rate of the model drops noticeably compared with the desktop platform due to the constraints of edge hardware computing power and scheduling mechanisms. There remains room to optimize real-time inference efficiency, and the model cannot meet the requirements of dynamic real-time monitoring at ultra-high frame rates.

Future research can be carried out to address the above limitations and improve the model performance. First, the dataset diversity will be significantly expanded, focusing on supplementing hard samples including color transition fruits with blurred feature boundaries, heavily occluded and overlapping fruits, and orchard images under extreme weather conditions. Diversified data augmentation strategies will be adopted to enhance the model’s feature discrimination ability for transitional maturity fruits and background interference targets, so as to fundamentally reduce the misclassification and missed detection rates of difficult samples. Second, lighter feature enhancement and adaptive feature fusion strategies will be introduced. Under the premise of maintaining detection accuracy, the computational overhead of the model will be further compressed to boost the inference speed and stability on embedded hardware, which adapts the model to high-speed dynamic field detection scenarios. Third, combined with practical agricultural production demands, the detection model can be integrated with auxiliary functions including fruit counting, ripeness-based yield statistics and intelligent picking linkage. An integrated intelligent agricultural monitoring system covering detection, data statistics and on-site application will be constructed to expand the practical application value and deployment scenarios of the proposed model.

## 5 Conclusions

Aiming at the prominent challenges in fig ripeness detection within protected greenhouses, including drastic variations in target scales, occluded and overlapping fruits, complex background interference, and limited computing power of edge devices, this study constructs a lightweight detection model YOLO12-SDS based on YOLO12n. The SPDSPPF module is adopted to boost the feature extraction performance for tiny targets and high-level semantic features. The learnable DySample upsampling module optimizes the reconstruction of fruit surface texture details. Furthermore, the SCSA attention mechanism is embedded into the P3 detection branch to suppress background noise and strengthen the feature representation related to fruit ripeness.

Experimental results demonstrate that the proposed model achieves remarkable accuracy improvements compared with the original YOLO12n. Its Precision, Recall, mAP@50 and mAP@50:95 reach 81.7%, 80.7%, 87.9% and 71.5%, respectively. Ablation experiments verify the synergistic performance gains brought by each improved module, and embedding the SCSA module into the shallow P3 feature layer yields the optimal detection performance. Compared with multiple mainstream lightweight detection models, YOLO12-SDS achieves superior overall detection performance. With a parameter count of 2.74 M, computational overhead of 6.0 GFLOPs and an inference speed of 115.6 FPS on desktop hardware, the model realizes an effective trade-off among detection accuracy, lightweight property and real-time performance. This study further completes the deployment and practical measurement validation of the model on the Jetson Orin Nano SUPER 8 GB embedded platform. The average single-frame inference latency on this edge device is only 9.4 ms, which can steadily satisfy the requirements of real-time field detection. This work provides reliable technical support for the field application of intelligent ripeness detection of figs in protected greenhouses.

The YOLO12-SDS model constructed in this study balances detection accuracy, anti-interference capacity under complex scenes and edge deployment advantages. It can effectively meet the real-time ripeness detection requirements of figs in protected greenhouses and deliver technical support for intelligent fruit grading and precise orchard management. Nevertheless, the generalization ability of the model under extreme weather and severe occlusion conditions, as well as its edge inference performance, still requires further optimization. Future work will expand datasets with complex hard samples and streamline the model architecture to improve robustness and real-time performance. Moreover, auxiliary functions including yield statistics and intelligent picking linkage will be integrated to establish an all-in-one intelligent monitoring system, so as to further enhance the field application and industrial promotion value of the proposed model.

## Data Availability

The raw data supporting the conclusions of this article will be made available by the authors, without undue reservation.
